# Dr. Vivian Pinn: a woman pioneer & leader

**DOI:** 10.1186/s40695-021-00070-7

**Published:** 2021-12-05

**Authors:** Gloria Bachmann, Nancy Woods

**Affiliations:** 1 Women’s Health Institute, Rutgers Robert Wood Johnson Medical School, New Brunswick, NJ USA; 2 University of Washington School of Medicine, Seattle, Washington USA

**Keywords:** Women’s health research, Dr. Pinn, Early pioneer advocate

## Abstract

The Women’s Health Institute in collaboration with the Journal of Women’s Midlife Health hosted a national roundtable with Dr. Vivian Pinn via Zoom to honor her for her achievements in the areas of women’s health, wellness, and research. The panelists included Gloria A. Bachmann, MD, MMS, Sherri-Ann Burnett-Bowie, MD, MPH, and Sioban D. Harlow, PhD.

This roundtable was in honor of Dr. Vivian Pinn, an early pioneer and advocate for women’s health and women’s health research. She was the first permanent Director of the Office of Research on Women’s Health (ORWH) at the National Institutes of Health, and the first woman in the United States to chair an academic pathology department at Howard University College of Medicine. She is now a senior scientist emeritus at the NIH Fogarty International Center, a Center which advances the NIH mission by supporting international health research to address global health needs.

During her tenure as Director of the Office of Research on Women’s Health (ORWH), Dr. Pinn not only promoted development of agendas to guide women’s health research, but also advocated for attention to the intersection of race/ethnicity and gender, factors impacting women’s health. She created opportunities for diverse groups of women to convene to develop inclusive research agendas, sensitive to health problems that had not been adequately studied. Finally, she led a variety of initiatives to enhance the training of research scientists in women’s health and sex and gender differences in health.

Dr. Pinn led development of women’s health research from an inclusive position: “If we were going to address including women in clinical studies and if we were going to address the health needs of women, we needed to promote opportunities for women to participate in directing and conducting that research.” She commented that it “was important to me to be able to address factors of diversity, not just racial diversity, but all different aspects of diversity” and that it was extremely important to “include diversity in inclusion policies to make sure we were addressing women and minorities in clinical trials.” Dr. Pinn also pointed out that women are playing influential roles across the life span, so it was necessary to not only expand diversity issues, but lifespan issues. At the same time, considering “women’s reproductive health issues that were not being addressed” was essential.

Aging processes women experience also warranted increased attention. Dr. Pinn pointed out how few studies of menopause had been completed when she joined the Office of Research on Women’s Health as Director, but with emphasis on midlife and menopause there were soon over 500 studies related to menopause in the NIH portfolio. She also helped change the bias of some scientists about women from diverse populations, e.g. African Americans, being unwilling to participate in studies. She mentioned the Women’s Health Initiative Study (WHI) and the Study of Women’s Health Across the Nation (SWAN) including diverse populations of women by design. She addressed the importance of SWAN, the largest longitudinal study of the transition from pre- to post-menopause in a multiracial multiethnic cohort, that is providing important information addressing many women’s questions. She outlined the importance of messages to researchers about NIH’s intent to study diverse populations of women, followed by a real effort to do that and a demonstration that women could be recruited from diverse populations.

Dr. Pinn brought perspectives to NIH from her work outside of government: among these were inclusivity in convening advisors who “were not within the Beltway” to have an opportunity to help influence a national research agenda. “Each of our series of strategic plan meetings … followed a format … with public hearings.” This strategy enabled women’s voices from many racial/ethnic and social groups from regions across the nation to be heard and woven into the tapestry of the research agendas. Testimony from these hearings was documented and informed the discussions of the agendas.

Dr. Pinn commented on the importance of “having more women representing those diverse populations being involved in the research because they understood the importance of reaching out to their populations or their communities to have them included.” She emphasized that having diverse populations represented in research was not only essential to obtain funding, but “was important so that we could address the health of women and understand why there were variations in outcomes, variations in responses to therapeutic interventions, or prevention strategies.”

Commenting on the research agendas for women’s health, Dr. Pinn indicated that the priorities shaped what ORWH could fund, while also allowing emphasis of topics beyond diseases, but also “wellness and prevention.” She also commented on the value of the ORWH being located in the Office of the NIH Director. Women’s health belonged to all of the institutes and ORWH engaged all in implementing the agenda. This location also provided visibility for the ideas advanced by diverse populations of women who helped set the agenda.

When asked about the pandemic and consequences for women’s health, Dr. Pinn indicated that many are now recognizing for the first time that “health disparities exist.” Differences in health status that were not appreciated earlier are now evident: “the statistics related to both impact and mortality and morbidity related to COVID-19 help to bring that out.” In addition, consideration of the stresses women (and men) and those in the health professions experience while caring for patients with COVID is growing. “This (COVID pandemic) is impacting women even more (than men) because of their child care or family care responsibilities.” Adapting to the pandemic is affecting women’s careers, especially those in research because of their family care and childcare responsibilities. Some have published recommendations for federal initiatives to provide support for child-care services for women in the workforce. This support may be especially important for women in scientific careers … and for men who also are providing care for their children. Dr. Pinn elaborated on the effects of the pandemic on mental health issues, not only for women and men, but for children, whose lives have been influenced by school closures.

In response to questions about racism, Dr. Pinn suggested that racism might be considered among social determinants of health. Her admonition “you need to walk in my shoes” seemed appropriate for those who may not understand current anti-racism protests. She would add sexism and homophobia along with many other isms to this list!

In relation to COVID vaccine use, Dr. Pinn suggested that populations that may be suspicious or mistrustful of vaccines would be encouraged to become immunized if we could say that “there were participants in the study like you and me. And so we know that the research that was done …can be interpreted as being applicable to populations that include us.”

In consideration of health research needs for aging women, Dr. Pinn identified the challenges of caregiving for family members, especially those with dementia. She emphasized the need for research to “prevent the effects of chronic disease on those who are at an advanced age”.

She eloquently described how the unique needs of women historically were not addressed in clinical trials: in fact, their health needs were not even on the radar. Most clinical trials in the past studied men, and therefore outcomes were based on men only. Past thinking was that ‘women are small men.’ Dr. Pinn pushed for more inclusion of women in clinical trials and for a proportion of clinical trials done that specifically addressed women’s issues. She wanted all women, including minorities, included, such that outcomes data addressed all women. Also she pushed for clinical trials to be commenced that evaluated issues such as menstruation, dysmenorrhea, pregnancy and menopause. Dr. Pinn promoted these initiatives when there were few other women doing this, or for that matter, when there were few women in leadership positions.

As Dr. Pinn succinctly noted, when she began as the Director of the Women’s Health Branch of NIH, “Our budget began to increase and we began to give more attention to what needed to be funded, as well as to expand in the areas of women’s careers. I felt very strongly that if we were going to address issues, such as women in clinical studies and if we were going to be addressing the health needs of women, we needed to also promote opportunities for women to participate in directing and conducting that research.”

She further went on to explain that these initiatives were critical, “…to make sure that we recognize that just as women were not the same as men, not all women are the same. And of course, that was important to me to be able to show that we were going to be able to address factors of diversity and not just racial diversity, but all different aspects of diversity.” She went on to explain that that inclusion policies had to address women from all cultures, races, and religions, in clinical trials. She wanted to be sure that research, especially NIH research was addressing the health of all women. She then added, that it also was important “…to look at life span issues.”

One of the main initiatives she led was changing the image of menopause and bringing more research and treatment to this time in a woman’s life, especially because women are living longer and issues that diminish their quality of life must be addressed. Also, she noted that an important aspect of aging, use of menopause and hormonal therapy and breast cancer. Before the Women’s Health Initiative (WHI), there were many unanswered questions regarding breast cancer, regardless of whether a woman was using or not using hormonal therapy. She said that from the start of this monumental women’s study, it was important to not only study these unanswered questions, but also to set a standard that all clinical trials must see women, not only as different from men, but each woman, herself, having unique health and wellness differences. Another important study delving into women’s issues is the SWAN study. Not only are the results important, but this study also exemplified that diverse groups of women are willing to participate in these types of studies.

Dr. Pinn promoted the importance of women’s health to the NIH and also advocated for diversity among the NIH/national leaders. These leadership positions included women who were advocates, scientists, nurses, physicians, physical therapists, administrators and dentists, to make sure we had full involvement across the health spectrum in order to execute their research agenda. Notably she helped establish this requirement. That is, if the study excluded women and minorities, it would not be NIH-funded. This phrase would be worked in to the NIH Revitalization Act of 1993, emphasizing that women and minorities must be included.

Currently, Dr. Pinn continues to play a prominent role in women’s health and continues to advocate in current health crises, such as the COVID-19 pandemic. She noted that possibly after this pandemic, the next health care crisis may be mental health issues in men, women and children. When Dr. Pinn was asked to address health disparities related to racism, she stated that “we aren’t really born with those concepts, … it is what we are exposed to.”

In concluding remarks, Dr. Pinn stressed the importance of preventing the effects of chronic disease, noting that as a population we are living longer and that most women are reaching their postmenopausal years. That makes all aspects of prevention as important as disease management. As she noted, “health should be wellness, not just being able to treat disease”.

This roundtable was attended by a nationally diverse audience, with many attendees being women at the beginning of their careers in a health care field.

## Data Availability

Not a research study.

